# Visual navigation technology for autonomous driving robots based on strategic gradient-REINFORCE algorithm

**DOI:** 10.1371/journal.pone.0347775

**Published:** 2026-05-11

**Authors:** Yuanyuan Hu

**Affiliations:** School of Electronic Information Engineering, Henan Institute of Information Science and Technology, Hebi, Henan, China; Beijing Institute of Technology, CHINA

## Abstract

Currently, autonomous driving robots face challenges of insufficient environmental perception and decision delay in visual navigation. To optimize the visual navigation performance of an autonomous driving robot under intricate working conditions, the paper optimizes the REINFORCE algorithm via integrating strategic gradients. A more efficient and precise visual navigation model built on the improved REINFORCE is designed. Experimental results demonstrate that the research algorithm’s accuracy reaches 95.7%, with 91.2% recall, superior to comparative algorithms. The improved algorithm has 92.5% navigation success rate in simulated environments, 15.8% surpassed than traditional methods. In real-world testing, the robot’s navigation decision time is reduced by 20.3%. Research algorithms can strengthen the visual navigation performance and offer a novel navigation strategy for autonomous driving robots, thereby promoting the application of autonomous driving technology.

## 1. Introduction

The growth of artificial intelligence technology has made Autonomous Driving Robots (ADRs) a vital research direction in the present technological field. ADR has shown broad application prospects in logistics distribution, environmental monitoring, and security patrol. One of its core functions is Visual Navigation (VN) technology [1]. VN simulates human visual perception ability, enabling robots to autonomously plan paths, avoid obstacles, and complete tasks in complex environments. Nevertheless, ADR still faces issues in VN. Firstly, the dynamic changes and diverse scenes in complex conditions pose extremely high requirements on the environmental perception capacity of robots. Secondly, the demand for real-time decision-making takes the efficiency and accuracy of navigation algorithms as a key bottleneck [2–3]. Conventional navigation approaches often exhibit issues, including insufficient adaptability and decision delays when facing complex scenarios, making it hard to cater to the needs of practical applications. Reinforcement Learning (RL) effectively addresses uncertainty in complex environments through learning optimal strategies through interaction between agents and the environment. The Strategic Gradient Algorithm (SGA) has been applied in VN tasks [4–5]. However, there are still drawbacks to in-depth exploration in existing research on how to optimize SGA to improve its performance in intricate dynamic environments. For instance, how to achieve efficient model training under limited data conditions, enhance the model’s ability to capture key visual features, and improve the real-time and robustness of algorithms, is still a difficulty of current research [6–7]. Given the above issues, the paper builds a REINFORCE algorithm based on a strategic gradient. By introducing the Actor-Critic Architecture (ACA) and combining the Residual Block (ResBlock) framework and Attention Mechanism (AM), an efficient ADR-VN model is constructed. This model aims to perfect the VN efficacy of ADR in intricate environments, enhance its adaptability to dynamic environments, and provide new solutions for the ADR-VN technology’s development.

The innovations of this study are: (1) Theoretical innovation: Based on the standard ACA, an optimized design is carried out, allowing the Critic network to share the feature extraction layer with the Actor network, and using the state value function as the benchmark. This design further optimizes the gradient propagation path based on the existing technology of parameter sharing in RL, significantly reducing the variance of the policy gradient, and enriching the theoretical application system of policy gradient optimization in VN in RL. (2) Technical innovation: By embedding attention modules after each group of three consecutive ResBlocks, a residual group attention cascade unit is designed to enable AM to focus on high-level semantic features; The visual front-end is lightweight through depth-separable convolution and channel pruning, which improves the efficiency of key feature capture and meets the real-time reasoning requirements of embedded platforms. (3) Application innovation: The improved algorithm can still maintain a high Navigation Success Rate (NSR) in complex scenarios such as variable lighting, dense obstacles, and extreme weather, and has excellent adaptability and robustness in both simulated and real environments; The improved algorithm provides practical technical solutions for ADR’s VN in a variety of practical application scenarios.

The contributions of this study are: (1) A lightweight end-to-end VN framework is developed, tailored for resource-constrained embedded platforms; Depthwise separable convolution and channel pruning are integrated to enable real-time inference without sacrificing performance. (2) In highly dynamic and visually challenging environments, a 92.5% NSR and sub-second decision-making latency are achieved. (3) It provides a replicable modular architecture that bridges the gap between theoretical RL progress and practical deployment, and provides a reference design for efficient vision-based autonomous navigation systems in real-world applications.

## 2. Related work

ADR-VN technology relies heavily on efficient environmental perception, real-time decision-making, and adaptive model training, with Deep Reinforcement Learning (DRL) being a key technical support to address complex scene challenges. Current research still faces bottlenecks in cross-domain knowledge integration, dynamic environmental adaptation, data utilization efficiency, and limited data training, which restrict the further improvement of navigation performance.

DRL-based mobile robot navigation mainly relies on single-modal visual perception for local navigation. Multi-modal visual fusion global navigation technology is still immature, and there is a problem of insufficient navigation safety in complex environments. In view of this, Z. Li et al. proposed a loop-deductive deep learning model for multi-modal visual robot navigation. This model integrated a cyclic reasoning mechanism to assist policy learning and combined scene recognition, semantic segmentation, and pose estimation information to achieve global decision-making. Simulation and real-scene tests showed that this model outperformed mainstream RL methods in terms of driving stability and safety. The collision rate of unmanned vehicles’ global decision-making decreased from 0.2% in the training state to 0.0% in the testing state. However, it did not involve lightweight model design, making it difficult to adapt to embedded platforms [8]. In response to the practical gap faced by unmanned aerial vehicles in autonomous navigation in complex unknown environments, as well as the low utilization rate of perception resources, P. Yue et al. proposed a semantic-driven autonomous VN method for unmanned aerial vehicles. This method combined transfer RL with end-to-end map-free VN, built a model including real reconstruction and motion decision-making, and generated navigation decisions through a spatio-temporal AM. The results showed that this method enabled unmanned aerial vehicles to achieve collision-free autonomous navigation without global information and unknown target positions. This model could be directly transferred to complex scenarios such as dynamic targets and multiple obstacles, without the need for re-training, but did not optimize for capturing key features in dynamic environments [9]. To solve the problems of difficulty in balancing obstacle avoidance and speed, and insufficient generalization ability in new environments for mobile robots’ autonomous navigation in static obstacles and dense pedestrian areas, Z. Xie and P. Dames proposed a new control strategy based on RL. This strategy integrated historical data from LiDAR, pedestrian kinematics data, and sub-object points as input, and was trained through a function with speed obstacle-related rewards. This strategy had higher NSR and faster average speed than mainstream algorithms, and could be adapted to different population sizes, unknown environments, and other robot platforms without re-training, but did not integrate an efficient feature extraction mechanism at the visual front end [10]. Mobile robot path planning has problems with insufficient navigation effectiveness and collision-free motion guarantees, and the lack of surrounding environment information leads to challenges in dynamic path planning in unknown environments. Therefore, K. Yeom proposed a collision-free path planning architecture based on DRL. This architecture did not require supervision, and the robot could perceive the unknown environment through DRL and use the predicted values of the control parameters output by it as the input for the next moment. Experimental results showed that compared with mainstream methods, this architecture could successfully solve complex navigation problems in dynamic environments. However, it still had room for improvement in navigation accuracy and decision real-time performance [11].

To address the challenges of difficult dynamic road obstacle detection and classification in autonomous driving, which can easily lead to collision accidents, H. Assemlali et al. proposed a deep learning-driven detection and classification method. They integrated data from multiple datasets to optimize the Convolutional Neural Network (CNN) architecture, enabling the identification of dynamic obstacles such as pedestrians, vehicles, and animals. This method demonstrated improved performance in various scenarios, with a classification accuracy of 99.5% and a detection accuracy of 97.1%. However, the paper did not mention the adaptability and real-time performance of this architecture in the actual navigation of autonomous robots [12]. To solve the conflict problem caused by aircraft failing to maintain the designated flight altitude at intersection points in urban air traffic, N. Yahi et al. proposed a distributed conflict detection and resolution method. They established an information interaction system for aerospace traffic entities, designed a rolling time-domain trajectory planning strategy, and combined the aircraft model and constraints to optimize the trajectory. Simulation verified that this framework can generate conflict avoidance strategies in real time, enabling safe flight at intervals at intersection points. However, the paper did not conduct practical scenario tests to validate the feasibility of the method [13]. Regarding the difficulty in detecting robot cutting points and the poor positioning accuracy, H. Wang et al. proposed the Fcaf3d network model. They combined the point cloud data collected by the time-of-flight camera and integrated an AM module to optimize feature extraction. Experimental results showed that the model achieved an F1 score of 88.57% on the test set, with good detection accuracy in different occlusion scenarios and small positioning errors. However, the paper did not verify the model’s adaptability in other fields [14].

Recent advanced deep reinforcement learning methods have distinct technical focuses and performance shortcomings in the field of visual navigation for autonomous robots: Proximal Policy Optimization (PPO) achieves stability in policy updates through trust region pruning, but it does not design a dedicated feature extraction module for the visual navigation task of autonomous robots, making it difficult to efficiently capture high-level semantic features in dynamic environments [15]. Soft Actor-Critic (SAC) enhances exploration capabilities in unknown environments through maximum entropy reinforcement learning, but its multi-network architecture design incurs high computational costs and is difficult to meet the real-time inference requirements of embedded platforms [16]. Deep reinforcement learning methods based on Transformer achieve global visual feature modeling through multi-head attention mechanisms, but the high computational complexity and large parameter volume of self-attention mechanisms lead to high inference latency and high hardware resource consumption, and these methods do not focus on lightweight design and lack targeted attention to key features of navigation scenarios [17]. In contrast, the improved REINFORCE algorithm proposed in this paper takes policy gradient optimization as the core, designs a shared feature extraction layer in the actor-critic architecture, builds a residual group attention cascading unit, and combines deep separable convolution and channel pruning at the visual front end, forming a full-process collaborative optimization design. Compared with the above advanced methods, the improved algorithm not only compensates for the shortcomings of single-dimensional optimization, but also simultaneously meets the requirements of efficient perception in complex dynamic environments, real-time inference on embedded platforms, and precise decision-making with limited data, achieving a coordinated improvement in perception accuracy, decision speed, and deployment adaptability.

## 3. Methods and materials

This section explores the optimization of REINFORCE based on strategic gradient and its application in ADR-VN by introducing the ACA to strengthen the performance and navigation efficiency. Meanwhile, an efficient improved model is constructed by combining ResBlock and AM to process complex visual information. By reducing the redundant parameters of the neural network and adopting depth-separable convolution and other techniques, the computational complexity and memory usage are lowered while ensuring its performance, ensuring that the algorithm can run in real time on the embedded computing platform carried by the robot.

### 3.1. Optimization of reinforce algorithm incorporating strategic gradient

The REINFORCE tries to maximize Cumulative Rewards (CRs). In ADR-VN tasks, robots need to choose the optimal action based on visual input and current state. Strategic Gradient refers to a gradient calculation method in RL where the parameters of the policy function are directly optimized to maximize the CR. Its classic mathematical definition is shown in equation (1).


$$\nabla_{\theta }J\left(\theta \right)={𝔼}_{\pi_{\theta }}\left[\overset{T}{\underset{t-0}{\sum }}\nabla_{\theta }log\pi_{\theta }\left(a_{t}|s_{t}\right)G_{t}\right]$$ (1)

In equation (1), ${𝔼}_{\pi_{\theta }}[\cdot ]$ calculates the mathematical expectation of all the trajectories generated by strategy $\pi_{\theta }$. τ is a complete trajectory, $\nabla_{\theta }J\left(\theta \right)$ is the strategy gradient, and θ represents the strategy parameter. $\pi_{\theta }\left(a_{t}|s_{t}\right)$ represents the probability of choosing action $a_{t}$ under the state $s_{t}$. $G_{t}$ is the CR at time step t. Compared with the standard policy gradient and REINFORCE methods, the proposed optimization policy gradient in this paper distinguishes itself in three key aspects: The classical policy gradient typically uses an independent value network as the benchmark, while this paper significantly reduces the gradient variance by designing a shared feature extraction layer in the ACA based on the state value function; The standard REINFORCE relies on complete trajectories to update parameters, while this paper combines the single-step update mechanism of Actor-Critic to improve training efficiency; Traditional policy gradient does not optimize for visual feature extraction, while this paper embeds the residual group – attention cascading unit in the visual front-end, making the gradient update more focused on high-dimensional semantic features. At the same time, through deep separable convolution and channel pruning, it achieves lightweighting and ensures the real-time inference capability of the embedded platform. The RGB images are normalized, and the pixel values are mapped to the [0,1] interval. The depth image is subjected to noise filtering and invalid value filling. Finally, features are extracted through CNN to obtain a visual feature vector with a dimension of 256. $s_{r}$ is the robot state, including the real-time position, speed, heading angle, and residual battery of the robot. After standardization, these state parameters are concatenated into a state feature vector with dimension 8 [18–20].

The reward function value of ADR-VN is based on the weighted importance of navigation tasks and statistical analysis of historical navigation data, and is quantitatively derived from the core task semantics and actual user needs of ADR-VN. The reward design follows the systematic principles of prioritizing the core task, constraining the safety bottom line, and assisting with efficiency optimization. The core task priority principle corresponds to the core needs of users using ADR to complete navigation tasks; The safety bottom line constraint principle corresponds to the strict requirements for collision-free operation of ADR in industrial scenarios; The efficiency-assisted optimization principle corresponds to the conventional requirements for ADR path stability and motion energy efficiency in practical applications. Based on these design principles, combined with the weight ratios of indicators such as task completion rate, collision occurrence rate, and path deviation rate in historical navigation data, specific reward values are derived. The terminal reward for successfully reaching the target (distance ≤ 0.3m) is set at + 100. Since reaching the target is the core task semantics of ADR-VN and the primary requirement of users for the navigation task, this value is 10 times the base reward unit, highlighting the core priority of task completion. The collision penalty (obstacle distance ≤ 0.5m, duration ≥ 0.5s) is set at −50. Since collision directly leads to the interruption of the navigation task and equipment damage, it is a strict safety risk to be avoided in industrial applications. This value is 50% of the core task reward and twice the general reward. Through a strong penalty mechanism, ADR’s obstacle avoidance behavior is strengthened, reflecting the hard constraint of the safety bottom line. The immediate reward of +2 for path adherence (deviation ≤ 0.2m) and penalty of −1 for deviation (>0.5m) are calibrated to balance path stability and movement flexibility. The ± 1 and ±0.5 rewards/penalties for speed control (1-3m/s) align with energy efficiency and motion safety constraints. A sensitivity analysis is conducted by adjusting each reward/penalty value by ±30%: results show that the model’s NSR fluctuates by less than 3%, and decision latency remains within 0.05s, confirming the robustness of the value settings. The interaction between multiple reward factors follows a hierarchical logic: safety (collision penalty) and task completion (target reward) are primary constraints, while path adherence and speed regulation act as secondary optimizations. When conflicts arise (e.g., path deviation to avoid obstacles), the negative penalty for collision (−50) outweighs the path deviation penalty (−1), guiding the model to prioritize safety. The CR formula begins with the core logic of RL: an ADR obtains an immediate reward $r_{k}$ at each time step k during navigation. Since future rewards are uncertain, a discount factor γ∈[0,1] is introduced to weight future rewards, balancing the model’s focus on short-term feedback and long-term navigation goals. The CR $G_{t}$ starting from time step t includes the immediate reward at t (weighted by $\gamma^{\text{0}}=1$) and discounted rewards from subsequent time steps, which is integrated into a continuous summation form until the navigation task terminates at time step T. Through controlled variable experiments, γ=0.99 is determined as the optimal value to ensure the model focuses on long-term target arrival without ignoring short-term actions like obstacle avoidance. The CR starts from time step t, as shown in equation (2).


$$G_{t}=\sum_{k=t}^{T}\gamma^{k-t}r\left(s_{k},a_{k}\right)$$ (2)

**Fig 1:**
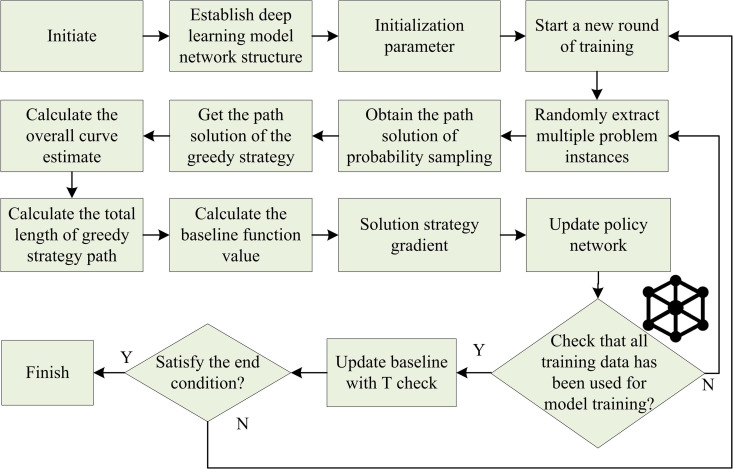
Flow of the REINFORCE algorithm.

In equation (2), $G_{t}$ is the CR. γ denotes the discount factor, r is the immediate reward, and k is the time step. The optimal value of γ is determined to be 0.99 through controlled variable experiments in the study. When γ = 0.99, the model can focus on the long-term navigation target without overly ignoring the immediate feedback of short-term actions. The specific process of the REINFORCE is shown in Fig 1.

In Fig 1, the policy network is initialized as CNN + fully connected layers: three convolutional layers (kernels 5 × 5, 3 × 3, 3 × 3; strides 2; outputs 16/32/64 channels) with two max-pooling layers (2 × 2), followed by three dense layers (256, 128, 6 neurons). The input is a 264-dimensional feature vector (composed of 256-dimensional visual features and 8-dimensional robot state features), and the basic output is 6 discrete actions, corresponding to straight driving, 15° left turn, 15° right turn, 30° left turn, 30° right turn, and braking. These six actions can be mapped to specific speed instructions for the left and right wheels of the robot. When driving straight, the speed of both wheels is the reference value. When turning, the speed of the single-side wheel decreases linearly according to the turning angle. When braking, the speed of both wheels is zero. At the same time, through the action space interpolation strategy, the discrete actions are smoothly mapped to continuous actions, supporting continuous adjustment of speed and steering angle. It can meet the requirements of smooth trajectory planning in high-dimensional action spaces. Parameters use the He normal initialization (bias = 0). Training starts with 1,000 diverse problem instances (straight, curved, multi-obstacle paths). Actions are sampled via roulette wheel from the policy’s probability distribution; A greedy path (max-probability actions) provides an upper-bound baseline. A moving average baseline based on CR is used to reduce policy gradient variance. Gradients are computed and parameters updated with Adam (initial lr = 1e-4, cosine annealing to 1e-5). Training stops when any of the following occurs: 100k iterations are reached, test success rate stabilizes (<1% fluctuation over 10 iterations), or gradient norm drops below 1e-5.

**Fig 2:**
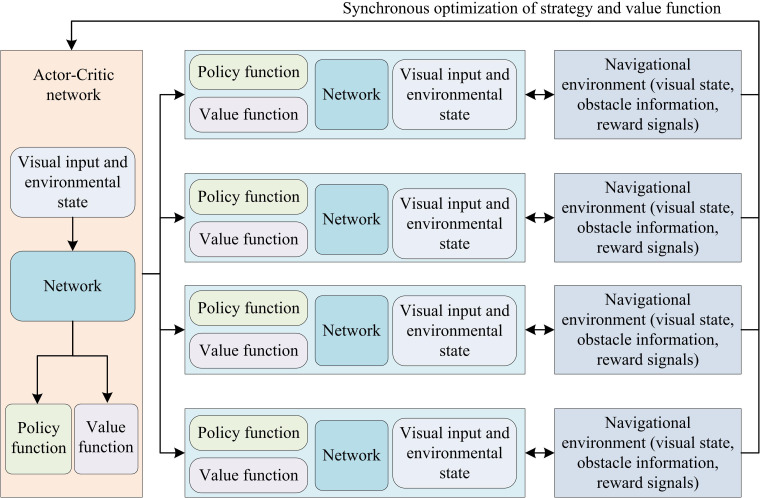
Schematic diagram of ACA in VN of ADRs.

This study introduces the Actor-Critic algorithm to optimize the REINFORCE algorithm, further optimizing its performance to improve navigation efficiency and stability. The Actor-Critic algorithm is an RL method that combines policy gradient and value function estimation. In ADR-VN, the Actor is responsible for generating actions, while Critic is responsible for evaluating the value of these actions [21–23]. The strategy gradient selection ACA algorithm is shown in Fig 2.

In Fig 2, the ACA network receives visual input and environmental state as inputs, and generates policy functions and value functions through its internal network structure. These functions are further used to guide the behavior decisions of robots in navigation environments. Each unit includes a strategy function, a value function, and a corresponding network structure. They synchronize optimization strategies and value functions to achieve more effective learning of optimal strategies. Each processing unit interacts with the navigation environment, receiving environmental feedback such as visual status, obstacle information, and reward signals, to continuously adjust and optimize its strategy and value evaluation, and improve the navigation performance of ADR in complex dynamic environments [24–25]. The ACA algorithm defines the state behavior value function as the expected CR that can be obtained by performing an action in a specific visual state, as given by equation (3).


$$Q^{\pi_{\theta }}\left(\phi \left(t\right),b_{i}\left(t\right)\right)=E_{\pi_{\theta }}\sum_{k=0}\left\{[\varepsilon^{k}r\left(t+k\right)|\left(\phi \left(t\right),b_{i}\left(t\right),\pi_{\theta }\right)]\right\}$$ (3)

In equation (3), $Q^{\pi_{\theta }}$ is the action-state value function. ε is the discount factor. ϕ(t) is the visual state at time t, including the robot’s position, speed, heading, and environmental information obtained through the camera. $b_{i}\left(t\right)$ is the action taken at t, such as steering angle, acceleration, etc. $\pi_{\theta }$ is the probability distribution of strategy, that is, action selection. r(t+k) is the immediate reward obtained at time (t+k), usually designed based on factors such as whether the robot successfully avoids obstacles and travels along a predetermined path [26]. The state value function represents the expected CR of the state at time t+1, updated as shown in equation (4).


$$v_{t+1}=y(\overset{T}{\underset{t}{\alpha }}v_{l}+\zeta_{t})$$ (4)

In equation (4), $v_{t+1}$ and $v_{t}$ are the state value function at time t+1 and t. $\overset{T}{\underset{t}{\alpha }}$ is the weight vector at t. $\zeta_{t}$ is the deviation at t. y is a function related to value updates, used to adjust value estimates. Deviation $\zeta_{t}$ is used to measure the difference between predicted and actual values, as shown in equation (5).


$$\zeta_{t}=r(t+1)+\varepsilon Q^{\pi_{\theta }}\left(\phi (t+1),b_{i}(t+1)\right)-Q^{\pi_{\theta }}\left(\phi \left(t\right),b_{i}\left(t\right)\right)$$ (5)

**Fig 3:**
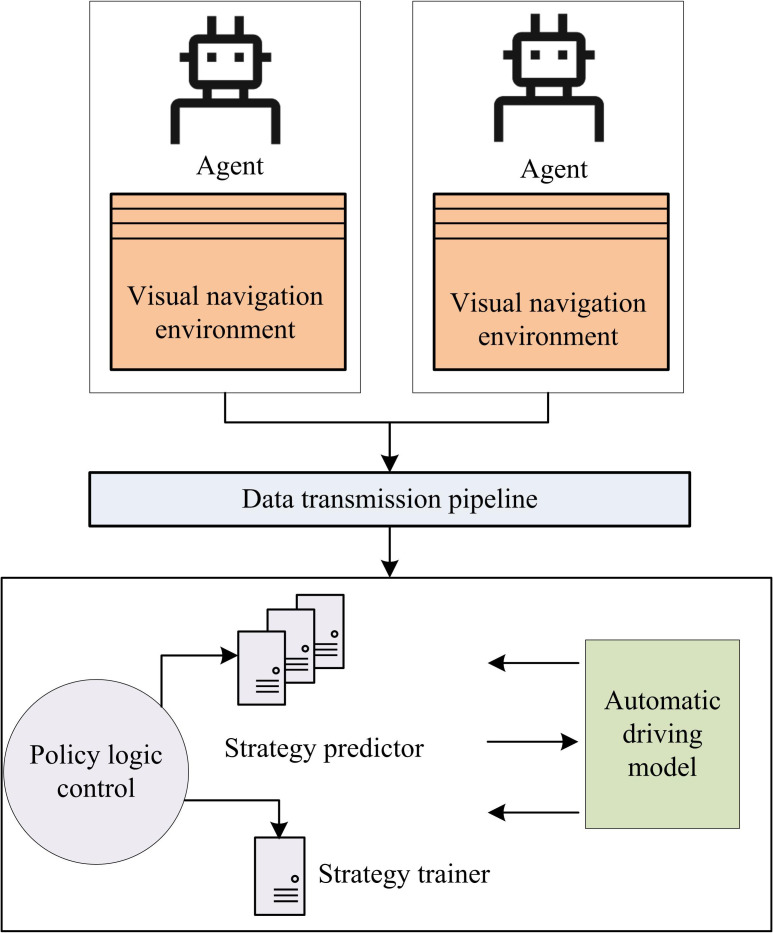
Parallel ACA for VN of an autonomous robot.

In equation (5), r(t+1) is the immediate reward obtained at time t+1. ε is a small positive number used to control the smoothness of deviations. $Q^{\pi_{\theta }}\left(\phi (t+1),b_{i}(t+1)\right)$ is the expected value in the new state. $Q^{\pi_{\theta }}\left(\phi \left(t\right),b_{i}\left(t\right)\right)$ is the expected value in the current state. The parallelized ACA for ADR-VN is shown in Fig 3.

**Fig 4:**
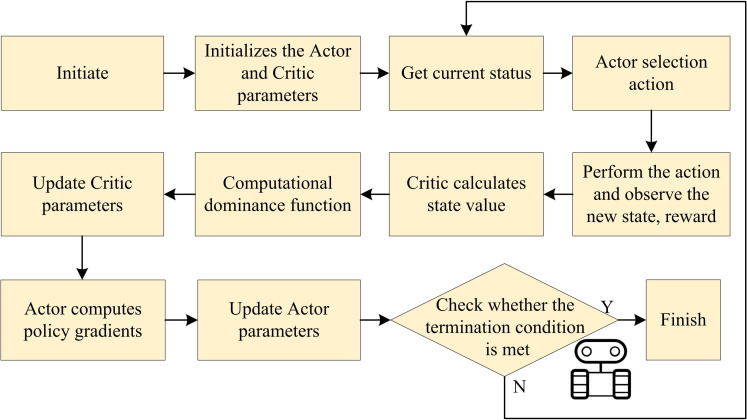
Flowchart of improved REINFORCE algorithm based on ACA.

In Fig 3, two intelligent agents are located in their respective VN environments and communicate with the policy logic control module through data transmission pipelines. The strategy logic control module includes a strategy predictor and a strategy trainer, which work together to optimize the autonomous driving model. The strategy predictor is responsible for predicting navigation strategies, while the strategy trainer updates model parameters based on the prediction results, thereby improving the VN ability of ADR [27–28]. The process of improving the REINFORCE algorithm based on ACA is shown in Fig 4.

In Fig 4, Actor and Critic networks are initialized with He normal and Xavier normal distributions, respectively, and biases set to 0.1 to avoid near-zero initial outputs. The system then acquires the current state via synchronized sensor data, visual input at 15 fps, and robot state at 50 Hz. The Actor selects actions using an ε-greedy strategy, which are executed via the motion control module to produce motor commands. New states and rewards are collected in real time. The Critic estimates state values for both current and next states, computes the TD error, and uses it directly as the advantage signal to evaluate action quality. Critic parameters are updated via Adam (lr = 5e-5) by minimizing squared TD error, while Actor parameters are updated via gradient ascent on the policy gradient (same learning rate). The loop continues until termination conditions are met: reaching the goal, collision, timeout, or battery below 5%. This collaborative ACA operates within a REINFORCE-based policy optimization loop.

### 3.2. Construction of vn model based on improved reinforce

**Fig 5:**
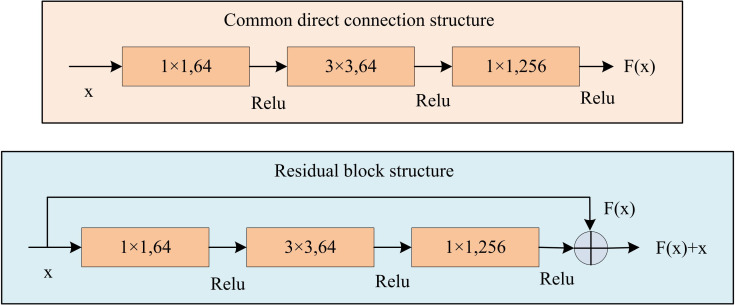
ResBlock structure diagram.

This paper effectively optimizes the strategic gradient of the REINFORCE by combining the ACA. This model needs to process complex visual information and make real-time decisions in dynamic environments, thus requiring higher performance of the algorithm. This study constructs an efficient VN model by integrating ResBlock and AM into an improved REINFORCE. ResBlock preserves input information through skip connections, allowing for constructing deeper network structures and improving model performance [29–31], as shown in Fig 5.

In Fig 5, the upper ordinary direct connection structure takes the feature map x as the input, which then passes through a convolutional layer with a kernel size of 1 × 1 and an output channel number of 64. After being nonlinearly transformed by the ReLU activation function, it is connected to another convolutional layer with a kernel size of 3 × 3 and an output channel number of 64. Similarly, after the ReLU activation, it finally passes through a convolutional layer with a kernel size of 1 × 1 and an output channel number of 256, and is activated by ReLU. Eventually, the feature map F(x) is output. The main path of the lower ResBlock structure is exactly the same as the ordinary direct connection structure. At the same time, an additional shortcut connection is added from the input x to the end addition operation. The original input feature x is added element-wise with the output F(x) of the main path, and the final output is F(x)+x. Through this residual connection design, while retaining the original feature information, effective deep feature extraction is achieved, which can alleviate the gradient disappearance problem that may occur during the training of deep neural networks. The design of ResBlocks allows the network to effectively avoid gradient problems when processing deeper features, improving the stability of network training [32–33]. The F(x), output after ResBlock processing, is shown in equation (6).


F(x)=ReLU(ReLU(3×3,64)(ReLU(1×1,64)(x)))+x (6)

**Fig 6:**
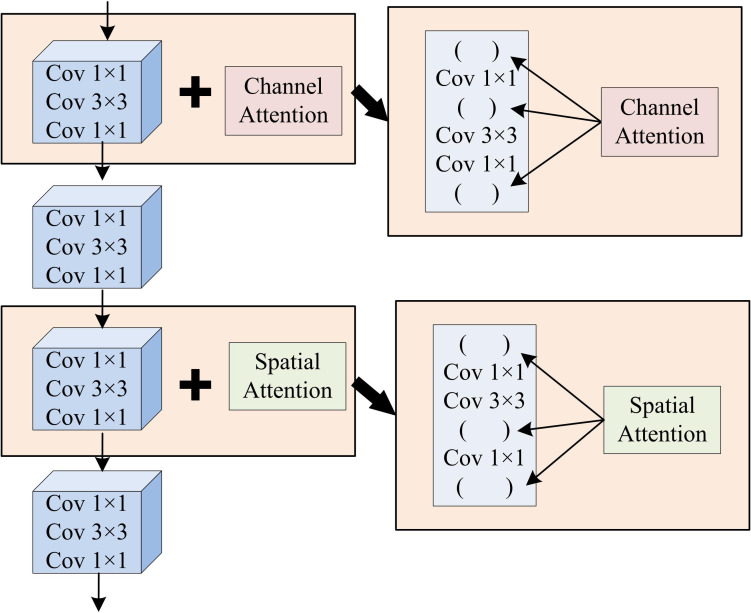
Module combining CAM and SAM.

In equation (6), x means the input feature data. ReLU is the ReLU function utilized to introduce nonlinear characteristics. AM further enhances the model’s ability to capture key visual features. The Channel Attention Module (CAM) and Spatial Attention Module (SAM) re-calibrate the channel and spatial feature response. This combination makes the model to focus more on important visual information, thereby rising the accuracy of navigation decisions [34–35]. Fig 6 shows the module structure combining CAM and SAM.

**Fig 7:**
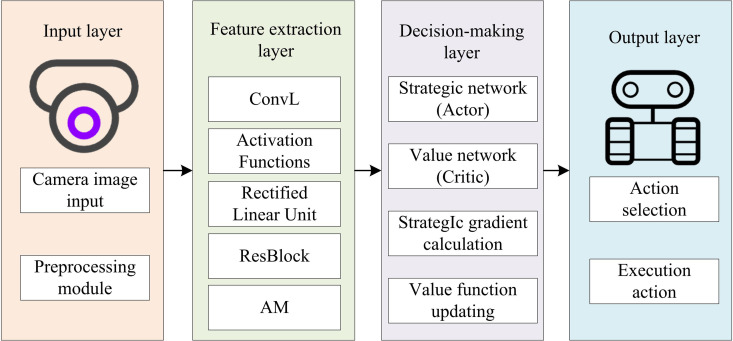
Diagram of the ADR-VN model based on the improved REINFORCE algorithm.

In Fig 6, the input feature map first enters the first-level processing unit, which contains parallel convolution branches and CAMs. The convolution branches consist of three different-sized convolution kernels of 1 × 1, 3 × 3, and 1 × 1. After the convolution operation completes, feature extraction is fused with the output of the CAM. The CAM re-weights the features extracted by the convolution in the channel dimension to enhance the feature information of key channels. The features after the first-level fusion continue to be passed downward to the second-level processing unit, whose structure is the same as the first level, retaining the parallel convolution branches composed of 1 × 1, 3 × 3, and 1 × 1 convolution kernels, while replacing them with a SAM. This module enhances the weights in the spatial dimension for the features extracted by the convolution, highlighting important spatial position features. Finally, the convolution branch of the secondary processing unit and the SAM output are fused, and the fused attention feature map is output after the final 1 × 1, 3 × 3, and 1 × 1 convolution kernel processing. Accurate enhancement and effective extraction of high-level visual features are achieved. This design strengthens navigation decisions by prioritizing informative environmental features [36]. The strategy network generates the probability distribution of actions built on the extracted features, while the value network assesses the current state’s value to assist the strategy network in making decisions. Eventually, the output layer outputs the final navigation action, as displayed in Fig 7.

The model in Fig 7 uses improved REINFORCE technology to process 15 fps sequences with optical flow to predict obstacle trends. Preprocessing normalizes, removes distortion/noise, and enhances illumination for feature consistency. The feature layer outputs a 256D vector via convolution, ResBlocks, and attention, extracting features from edges to semantics like obstacles/lane lines. The Actor outputs Softmax-normalized actions with regularization; The Critic outputs [0, 100] state values for TD errors. Actions selected via ε-greedy are sent via ROS to the chassis controller and converted to wheel speeds using a differential model. Training uses asynchronous parallel updates and gradient clipping (threshold 10) for efficiency/stability.

## 4. Results

This study comprehensively evaluates the performance of the improved REINFORCE algorithm in ADR-VN tasks. Through experiments in simulated environments and real scenarios, the Accuracy (ACC), recall, convergence, runtime, and NSR of the algorithm are analyzed and compared with other algorithms. In addition, this study also examines the adaptability and decision-making efficiency of the algorithm in different environments to verify its navigation performance in complex dynamic environments.

### 4.1. Comprehensive performance analysis of improved reinforce algorithm

To further assess the performance of the improved REINFORCE in ADR-VN tasks, many experiments are performed. To simulate the driving environment in the real world, the CARLA environment is used for autonomous driving simulation. This environment provides a configurable virtual city map, including elements such as roads, traffic signals, pedestrians, and other vehicles. The experiment selects the Town05 map, which includes various scenes such as urban roads, highways, tunnels, and parking lots. The weather conditions are set to three types: sunny, rainy, and foggy, respectively simulating different lighting and visibility scenarios. The 1,000 problem instances generated in the experiment cover three typical scenarios: straight roads (30%), curves (40%), and areas with dense obstacles (30%). Each scenario undergoes systematic changes in terms of lighting (daytime strong light, nighttime weak light, sudden changes in tunnel brightness), dynamic obstacle density (0–5 moving objects), and path complexity (curvature radius of 2–10 m). The training set and test set are divided in an 8:2 ratio, and the test instances do not appear in the training set in terms of layout, obstacle type, and lighting conditions, ensuring out-of-distribution evaluation. All instances are independently generated through random seeds to avoid data leakage. All instances are independently generated through random seeds to prevent data leakage. To analyze the sample efficiency, quantitative tests are conducted on the core indicators of the model’s NSR and decision accuracy under different training rounds. The results show that when the training rounds are reduced to 70%, 50%, and 30% of the original setting, the model’s NSRs are 91.3%, 88.2%, and 82.5%, and the decision accuracies are 94.5%, 92.1%, and 87.6%, respectively. The performance shows a smooth decline as the training rounds decrease, without a sudden drop, demonstrating good sample utilization efficiency. Table 1 shows the experimental configuration.

**Table 1:** Experimental settings.

Parameter name	Parameter value
Operating system	Ubuntu 20.04 LTS
Python	3.8
PyTorch	1.8.1
CUDA	11.1
cuDNN version	8.0.5
CPU	Intel Xeon Platinum 8358
GPU	NVIDIA Tesla V100
Memory	256GB
Learning rate	0.0001
Discount factor	0.99
Batch size	1024
Maximum iterations	100000
Optimizer	Adam

**Fig 8:**
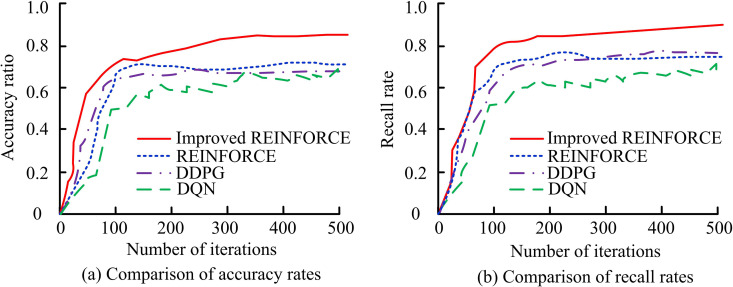
Comparison of ACC and recall rates of several algorithms.

The improved REINFORCE algorithm is compared with the REINFORCE algorithm, Deep Deterministic Policy Gradient (DDPG), and Deep Double Q-Network (DQN). The selection of the baseline algorithm strictly follows the principles of domain representativeness and task adaptability: The traditional DRL navigation method is selected as the basic comparison group, covering two core frameworks of early policy gradient and value iteration. Modern mainstream methods are included as advanced comparison groups, covering stability optimization, exploration ability enhancement, and global feature modeling, ensuring the coverage of typical algorithms of different technical paths in the ADR-VN domain. The parameters of each algorithm are all set to the optimal configuration verified in the original literature and unified the experimental environment parameters: REINFORCE’s learning rate: 1e-3, with discount factor of 0.98; DDPG experience replay buffer size: 1e6, target network update rate: 0.001; DQN dual network update interval: 1000 steps, ε decay rate: 0.995; PPO trust region clipping coefficient: 0.2, default learning rate: 3e-4 (after optimization for navigation task, it becomes 1.5e-4); SAC default temperature parameter: 0.2 (after optimization for navigation task, it becomes 0.15), discount factor: 0.99; Transformer-based VN methods (Transformer-DRL) number of multi-head attention heads: 8, feature dimension: 256. All algorithms are implemented based on the PyTorch framework, with 50,000 training iterations, batch size 64, and the physical parameters and sensor parameters of the experimental environment remain consistent to ensure the fairness of the comparison. The ACC and recall rates of several algorithms are shown in Fig 8.

**Fig 9:**
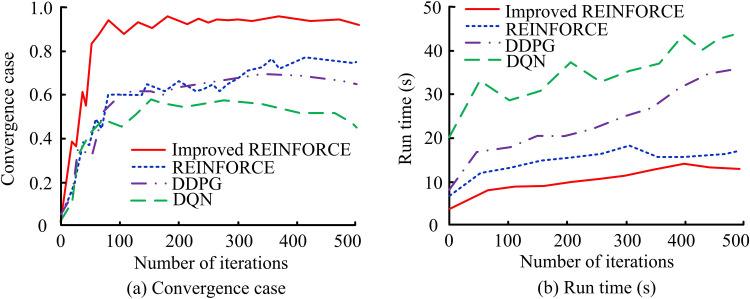
Comparison of convergence and runtime of several algorithms.

In Fig 8 (a), as the iterations increase, the ACC of the improved algorithm rapidly rises and reaches its highest ACC after approximately 300 iterations, approaching 0.95. In Fig 8 (b), the recall rate of the improved algorithm rapidly increases and reaches its highest point after approximately 400 iterations, approaching 0.91. The improved algorithm outperforms other algorithms in both ACC and recall metrics, demonstrating its faster learning and convergence ability during the iteration process. This shows that the improved algorithm has a higher NSR and recall rate in ADR-VN tasks, thereby improving overall navigation performance. The convergence and runtime comparison of several algorithms are shown in Fig 9.

**Fig 10:**
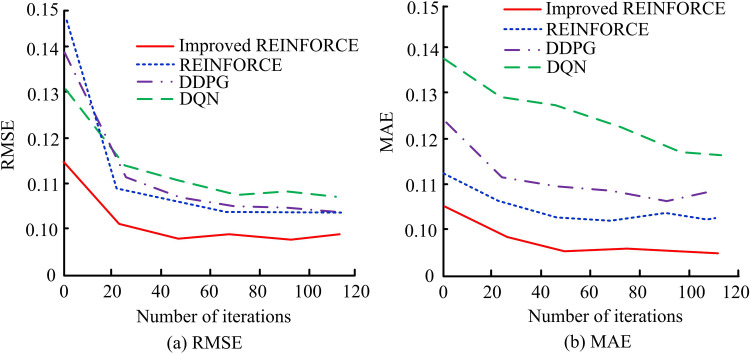
Comparison of RMSE and MAE of several algorithms.

In Fig 9 (a), the improved algorithm quickly converges when the number of iterations reaches about 100, with a convergence rate close to 1.0, demonstrating its efficiency during the training process. The REINFORCE converges at around 200 iterations, with a convergence rate of approximately 0.65. The convergence rates of DDPG and DQN reach approximately 0.6 and 0.5 at 300 and 400 iterations, indicating a slower convergence speed. The research algorithm has significantly better convergence speed than the compared algorithm, and can achieve higher convergence rates faster. In Fig 9 (b), the improved algorithm has the shortest runtime throughout the entire iteration process, gradually increasing from about 5 seconds to about 15 seconds from the beginning to 500 iterations. The runtime of the REINFORCE increases from about 10 seconds to about 20 seconds, DDPG increases from about 15 seconds to about 35 seconds, and DQN increases from about 30 seconds to about 45 seconds. The improved algorithm exhibits the best performance in ADR-VN tasks, with fast convergence speed and short runtime. This demonstrates that the algorithm can well improve the training and running efficiency of the model, and is suitable for application in autonomous driving scenarios that require fast response. The Root Mean Square Error (RMSE) and Mean Absolute Error (MAE) of several algorithms are shown in Fig 10.

**Fig 11:**
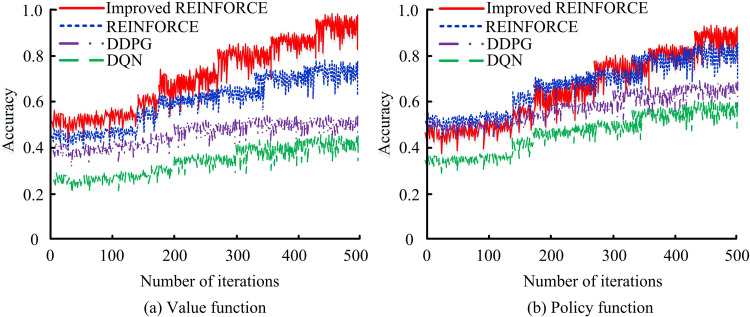
Accuracy of the algorithm in the value function and the policy function.

In Fig 10 (a), as the iterations rise, the RMSE value of the improved algorithm rapidly decreases and stabilizes after approximately 20 iterations, ultimately stabilizing at around 0.10. The improved algorithm performs the best in reducing RMSE, followed by REINFORCE and DDPG, while DQN performs relatively poorly. In Fig 10 (b), as the iteration grows, the MAE value of the research algorithm rapidly decreases and stabilizes after approximately 20 iterations, ultimately stabilizing at around 0.09. The improved algorithm outperforms the compared algorithm in reducing both RMSE and MAE error metrics, demonstrating faster convergence speed and higher accuracy during the iteration process. This shows that the improved algorithm has higher navigation accuracy and stability in ADR-VN tasks, which can improve overall navigation performance. The accuracy of the algorithm in the value function and strategy function is shown in Fig 11.

In Fig 11 (a), at the beginning of iteration, the accuracy of the value function of the improved algorithm rapidly increases, reaching over 0.8 after approximately 300 iterations, and maintaining stable growth in subsequent iterations, ultimately approaching 1.0. The improved algorithms perform best in value function evaluation, enabling faster and more accurate assessment of the value function, thereby improving decision quality. In Fig 11 (b), the improved algorithm also performs outstandingly in terms of the accuracy of the policy function, reaching over 0.8 after approximately 300 iterations and maintaining stable growth in subsequent iterations, ultimately approaching 1.0. The improved REINFORCE algorithm also performs the best in policy function evaluation, which can evaluate policy functions faster and more accurately, improving the accuracy and overall performance of policy selection.

### 4.2. Evaluation of adr-vn application effectiveness

**Fig 12:**
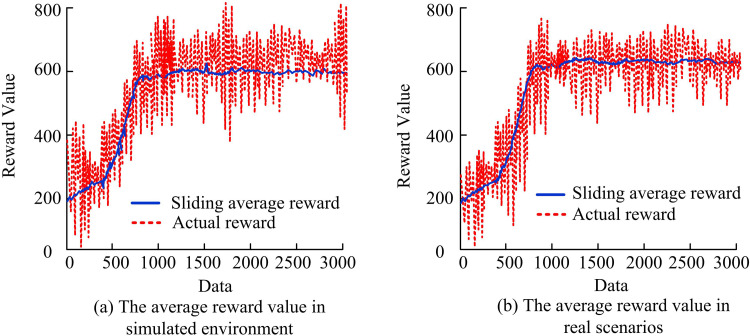
Average reward value of VN of autonomous robot in simulated environment and real scene.

To assess the practical application effect of the improved algorithm in ADR-VN tasks, the performance of the algorithm in simulated environments and real scenes is further examined. The average reward value of ADR-VN in the simulated environment and real scene is shown in Fig 12.

In Fig 12 (a), as the number of data points grows, the sliding average reward value gradually rises and tends to stabilize, while the actual reward value shows significant volatility. After approximately 1,000 data points, the sliding average reward value tends to stabilize, indicating that the model’s learning in the simulated environment gradually converges. However, although the actual reward value has shown an overall upward trend, it fluctuates greatly, indicating a performance difference between the simulated and real environments. In Fig 12 (b), in the real scene, the sliding average reward value also tends to stabilize after about 1,000 data points, but the average reward value is slightly lower than in the simulated environment. The actual reward value fluctuates more in real scenarios and shows a significant decrease at certain data points. This is due to the complexity and uncertainty in the real environment. Nevertheless, the overall trend shows an increase in reward values, indicating an improvement in performance in real-world environments. The improved REINFORCE has shown good learning performance in both simulated and real-world environments, achieving high average reward values with fewer data points and demonstrating good convergence and stability. The success rate of ADR-VN is evaluated in both simulated and actual environments, as listed in Table 2.

**Table 2:** NSR between simulated environment and real scenario.

Algorithm type	Simulated environment NSR (%)	Real world NSR (%)	Path deviation rate (%)	Navigation stability (%)
Improved REINFORCE	92.5	92.3	2.1	93.5
REINFORCE	90.1	90.5	3.1	86.1
DDPG	81.7	81.2	2.8	85.2
DQN	81.2	88.4	3.5	73.6

In Table 2, the improved REINFORCE achieves 92.5% NSR in simulated environments and 92.3% in real scenarios, with 2.1% path deviation rate and 93.5% navigation stability. The 7.7% of operation failures mainly stems from two situations: the failure of visual feature extraction under extreme lighting conditions and the lag in predicting sudden changes in dynamic obstacles. Among them, the former accounts for 62% and the latter accounts for 38%. The system is equipped with real-time fault detection and recovery mechanisms for such failure scenarios. It completes visual feature restoration through deep image distance information, predicts the movement trend of obstacles based on historical trajectories, and triggers temporary deceleration strategies. The average fault recovery time is 1.2 seconds, enabling a quick return to the normal navigation state. The success rates of REINFORCE in the two scenarios are 90.1% and 90.5%, with path deviation rate and navigation stability of 3.1% and 86.1%. The four indicators of DDPG are 81.7%, 81.2%, 2.8%, and 85.2%, while DQN is 81.2%, 88.4%, 3.5%, and 73.6%. The improved algorithm has high NSR in both simulated and real environments, demonstrating its superior performance in VN tasks. To comprehensively evaluate the performance in ADR-VN tasks, the Navigation Decision Time (NDT) in different scenes is analyzed, as exhibited in Table 3.

**Table 3:** NDT between simulated environment and real scenario.

Algorithm type	Improved REINFORCE	REINFORCE	DDPG	DQN
Simulated environment decision time (seconds)	0.85	1.0	1.15	1.05
Real scenario decision time (seconds)	0.68	0.8	0.86	0.87
Response time (seconds)	0.45	0.50	0.60	0.50
Path planning time (s)	0.35	0.40	0.25	0.30
Percentage reduction in decision time (%)	20.6	20.0	25.2	17.1

In Table 3, the improved algorithm has a decision time of 0.85 seconds in simulated environments, 0.68 seconds in real scenarios, a response time of 0.45 seconds, and a path planning time of 0.35 seconds. Compared to simulated environments, the decision time is reduced by 20.6%. The four values of the REINFORCE algorithm are 1.0 s, 0.8 s, 0.50 s, and 0.40 s, resulting in a 20.0% reduction in decision time. The various indicator times of DDPG and DQN algorithms are (1.15 s, 0.86 s, 0.60 s, 0.25 s) and (1.05 s, 0.87 s, 0.50 s, 0.30 s), reducing decision time by 25.2% and 17.1%. The improved algorithm has demonstrated faster decision times in both simulated environments and real scenarios, with the shortest decision time, response time, and path planning time being relatively short in real scenarios. This demonstrates the algorithm’s good adaptability and efficiency in real scenarios. To further verify the performance of the improved REINFORCE algorithm in extreme scenarios, the study adds extreme weather and complex obstacle tests in the CARLA simulation environment. The extreme weather scenarios are heavy rain (visibility ≤ 50m) and thick fog (visibility ≤ 30m). In CARLA, the precise simulation of extreme weather is achieved by adjusting the WeatherParameter set. In the heavy rain scenario, the precipitation intensity is set to 100.0, the size of raindrops is 0.2, and the speed of raindrops is 20.0. At the same time, the atmospheric scattering effect is enabled to simulate the refraction and obstruction of rainlight. In the thick fog scenario, the fog concentration is set to 0.8, and the attenuation coefficient is 0.05. The global fog effect and local atmospheric perspective are combined to reduce the environmental visibility. The sensor noise is modeled separately for the visual camera and the depth camera. For the visual camera, Gaussian noise (SNR = 20dB) and salt-and-pepper noise (density = 0.03) are added. For the depth camera, distance-related Gaussian noise (error increases linearly with the detection distance, with a maximum error ≤ 0.3 m) is added. This simulates the noise interference of sensors in the real environment. The complex obstacle scenarios include dense static obstacles (obstacle spacing ≤ 1m) and dynamic obstacles randomly changing lanes. The results are shown in Table 4.

**Table 4:** Comparison of Navigation Performance of various algorithms in Extreme Scenarios.

Algorithm type	Improved REINFORCE	REINFORCE	DDPG	DQN
Success rate of rainstorm scenes (%)	88.2	80.1	72.5	70.3
Success rate of thick fog scenes (%)	85.5	76.3	68.8	65.2
Success rate of dense static obstacles (%)	90.3	82.5	75.6	73.1
Success rate of dynamic obstacle lane changing (%)	89.7	81.2	73.8	71.5
Average fault recovery time (seconds)	1.2	2.5	3.1	3.5

In Table 4, the improved REINFORCE algorithm still maintains a high NSR in extreme scenarios: the success rate in the rainstorm scene is 88.2%, and it focuses on nearby obstacles and lane lines through image enhancement and AMs, reducing the interference of rainwater. The success rate in thick fog scenes is 85.5%. The distance information of depth images is utilized to assist in determining the position of obstacles, compensating for the insufficient visibility of visual images. The success rate in the dense static obstacle scene is 90.3%. Through the fine features extracted by the ResBlocks, the obstacle gaps are accurately identified, and the detour paths are planned. The success rate in the dynamic obstacle lane-changing scene is 89.7%. The movement trend of obstacles is analyzed through historical frames, and the navigation strategy is adjusted in advance. In addition, the average fault recovery time of the improved algorithm is 1.2 seconds. The fault recovery time refers to the time it takes for the robot to return to the normal navigation state after deviating from the path or approaching an obstacle. The improved algorithm achieves rapid recovery through rapid value assessment and strategy adjustment, which is much lower than that of other comparison algorithms, further verifying its robustness and practicality in complex and extreme scenarios. A special evaluation is conducted on the dynamic obstacle prediction method based on the optical flow algorithm adopted by the model. The results show that after enabling this method, the NSR in scenarios with dynamic obstacle changes increases by 9.2%, and the prediction error of the obstacle movement trend is controlled within 0.15 m. Compared with the basic model without enabling this method, the decision response in dynamic scenarios is 0.2 seconds earlier, effectively reducing the navigation failure caused by the lag in dynamic obstacle prediction.

To verify the reliability of the experimental results, a one-way analysis of variance (ANOVA) and post-hoc Tukey HSD test are conducted on the core performance indicators of each algorithm. The significance level is set at 0.05. The statistical analysis results are shown in Table 5.

**Table 5:** Statistical significance analysis results of performance indicators for each algorithm.

Performance indicators	F value	*p* value	REINFORCE	DDPG	DQN
Navigation success rate (%)	12.87	<0.001	*p*=0.032 (significant)	*p* < 0.001 (Extremely significant)	*p* < 0.001 (Extremely significant)
Decision time (s)	9.34	<0.01	*p*=0.041 (significant)	*p*=0.008 (Significant)	*p*=0.009 (Significant)
Accuracy rate (%)	15.62	<0.001	*p*=0.028 (significant)	*p* < 0.001 (Extremely significant)	*p* < 0.001 (Extremely significant)
Recall rate (%)	11.49	<0.001	*p*=0.035 (significant)	*p* < 0.001 (Extremely significant)	*p* < 0.001 (Extremely significant)

Note: The data is in comparison with the improved REINFORCE method.

The results show that there are statistically significant differences between the improved REINFORCE algorithm and the comparison algorithm in all core indicators, confirming the reliability of its performance advantage rather than being accidental. To clarify the independent contributions of ACA, ResBlock, and AM, ablation experiments are conducted. The performance of different module combinations is shown in Table 6.

**Table 6:** Performance comparison table of ablation experiments.

Module combination	Success rate of simulated environment navigation (%)	Decision time (s)	Accuracy rate (%)	Recall rate (%)	Path deviation rate (%)
Basic REINFORCE	82.6	1.12	89.3	83.5	4.8
REINFORCE+ACA	88.3	0.98	92.1	87.8	3.6
REINFORCE+ACA + ResBlock	90.5	0.91	94.2	89.7	2.7
REINFORCE+ACA + AM	89.1	0.95	93.5	88.4	3.2
REINFORCE+ACA + ResBlock+AM	92.5	0.85	95.7	91.2	2.1

Based on REINFORCE+ACA, merely introducing the residual structure can increase the NSR by 2.2%, the accuracy rate by 2.1%, and the path deviation rate by 0.9%. This verifies its core gain in extracting deep visual features. Introducing only the AM can increase the NSR by 0.8%, the accuracy rate by 1.4%, and the path deviation rate by 0.4%, demonstrating its additional effect of focusing on key semantic features. When the two are combined, performance is further enhanced. Compared with a single residual structure, the NSR increases by 2.0%, confirming that the deep feature extraction of residual structures complements the key feature selection of AM, rather than the independent effect of a single mechanism. To comprehensively verify the performance level of the improved algorithm in modern DRL methods, a comparative experiment is conducted by comparing it with PPO, SAC, and Transformer-DRL. The experimental environment and parameter settings are consistent with those in Table 1. The specific performance comparison results are shown in Table 7.

**Table 7:** Performance comparison of improved algorithm and modern DRL methods.

Algorithm type	Improved REINFORCE	PPO	SAC	Transformer-DRL	REINFORCE	DDPG	DQN
Success rate of simulated environment navigation (%)	92.5	89.7	88.5	90.2	90.1	81.7	81.2
Success rate of real scene navigation (%)	92.3	89.2	88.7	89.5	90.5	81.2	88.4
Decision time (s)	0.85	0.92	0.95	1.18	1.0	1.15	1.05
Accuracy rate (%)	95.7	93.4	92.8	94.1	89.3	87.6	86.9
Recall rate (%)	91.2	88.6	87.9	89.3	85.1	82.4	80.7
Path deviation rate (%)	2.1	2.5	2.7	2.3	3.1	2.8	3.5
Navigation stability (%)	93.5	90.3	89.6	91.2	86.1	85.2	73.6

The comparison results show that the improved REINFORCE algorithm outperforms other methods in all key performance indicators: the success rates of navigation in the simulated environment and the real scene reach 92.5% and 92.3%, respectively, which are 2.3–4.0% higher than those of PPO, SAC, and Transformer-DRL methods. The decision time is only 0.85 seconds, significantly shorter than 1.18 seconds of Transformer-DRL, making it more suitable for embedded platform requirements in terms of real-time performance. The accuracy and recall rate remain leading at 95.7% and 91.2%, respectively, the path deviation rate is as low as 2.1%, and the navigation stability reaches 93.5%, demonstrating a more balanced VN ability in complex environments. Its performance advantage stems from the collaborative optimization of the ACA, ResBlocks, and AM, as well as the lightweight visual front-end design that balances real-time performance and accuracy. To comprehensively verify the algorithm’s robustness under practical disturbance scenarios, cross-validation tests are conducted on the embedded platform (NVIDIA Jetson Xavier NX), covering variable kinematic states of the robot and sensor noise interference. The kinematic states include different speed ranges (0.5-2m/s, 2-4m/s) and motion modes (straight-line, continuous turning, start-stop alternating). Sensor noise includes Gaussian noise (SNR = 20dB/30dB) and salt-and-pepper noise (density = 0.01/0.03) added to visual input. The robustness is evaluated by averaging core performance indicators and resource consumption across all test scenarios. The test results are shown in Table 8 below.

**Table 8:** Cross-validation comparison of algorithm robustness and resource consumption.

Algorithm type	Average NSR across multiple scenarios (%)	Average inference latency across multiple scenarios (ms)	Average computing load (GPU utilization rate, %) across multiple scenarios	Stable memory usage across multiple scenarios (MB)	Total model parameters (M)	Average path deviation rate across multiple scenarios (%)
Improved REINFORCE	91.7	18.9	43.2	48.5	1.28	2.3
REINFORCE	88.2	24.1	52.5	66.1	2.13	3.2
DDPG	80.5	27.3	59.1	79.8	3.05	2.9
DQN	86.8	25.9	55.0	72.6	2.76	3.6
PPO	89.5	23.4	50.1	61.7	1.97	2.6
SAC	87.9	25.2	57.3	89.9	2.81	2.8
Transformer-DRL	89.8	34.2	74.0	127.2	8.64	2.4

Note: The multi-scenario average value is the mean of indicators measured under 8 test scenarios (2 speed ranges × 3 motion modes × 2 sensor noise levels, excluding redundant combinations).

As shown in Table 8, the improved REINFORCE algorithm demonstrates extremely high robustness in cross-validation tests: its multi-scenario average NSR reaches 91.7%, which is 3.5% higher than that of the original REINFORCE algorithm, 1% higher than DDPG, 2% higher than Transformer-DRL, and maintains high reliability even under changing motion states and sensor noise interference. In terms of real-time performance stability, the average inference delay across multiple scenarios is only 18.9 ms, with a fluctuation range of less than 0.6 ms for different scenarios, and the average computing load remains at 43.2%, indicating that the performance degradation caused by environmental and state changes is minimal. The stable memory usage remains at 48.5MB, and the average path deviation rate is controlled at 2.3%, indicating its strong resistance to sensor noise and adaptability to various motion modes. This cross-validation result confirms that this algorithm not only achieves a balance between performance and resource consumption under ideal conditions, but also remains stable and reliable in the face of common practical disturbances such as variable motion states and sensor noise, fully meeting the robustness requirements of ADR-VN in complex practical applications.

To quantitatively verify the computational efficiency and cross-platform deployment adaptability of the lightweight design of the improved REINFORCE algorithm, three mainstream embedded platforms in the current ADR-VN field are selected for the actual test (NVIDIA Jetson Nano, NVIDIA Jetson TX2, NVIDIA Jetson Xavier NX). The core computational efficiency and resource occupation indicators of the algorithm under each platform are simultaneously collected. All indicators are the average values obtained from multiple scenarios of testing. The actual test data are shown in Table 9.

**Table 9:** Comparison of algorithm deployment and computational efficiency on mainstream embedded platforms.

Embedded platform	Algorithm type	Average inference latency (ms)	Peak GPU utilization (%)	Stable memory footprint (MB)	Average power consumption (W)	GPU steady-state operating temperature (°C)
NVIDIA Jetson Nano	Traditional REINFORCE	58.7	92.5	78.6	8.3	68
	Improved REINFORCE	32.5	68.3	45.2	5.8	52
NVIDIA Jetson TX2	Traditional REINFORCE	41.3	79.8	75.4	13.7	62
	Improved REINFORCE	24.1	52.6	46.8	10.2	48
NVIDIA Jetson Xavier NX	Traditional REINFORCE	24.1	52.5	66.1	18.9	56
	Improved REINFORCE	18.9	43.2	48.5	15.5	45

In Table 9, the improved REINFORCE algorithm, with its lightweight design including deep separable convolution, achieves full-dimensional optimization on three embedded platforms: memory footprint ranges from 45.2 to 48.5MB, a reduction of 39.9% to 42.5% compared to traditional algorithms; peak GPU utilization drops by 17.7% to 26.2%, eliminating the problem of resource overload; inference latency reduces by 44.6% to 49.7%, with the entire platform’s latency under 60 ms, meeting real-time requirements; average power consumption decreases by 30.1% to 30.7%, all controlled within 16W, without any heat dissipation pressure; compared with traditional REINFORCE, the temperature is reduced by 14–16°C, demonstrating the thermal performance advantages of the lightweight design. The improved REINFORCE algorithm is adapted to different embedded platforms with varying computing capabilities, achieving stable optimization results and a dual improvement in computational efficiency and hardware compatibility. To verify the performance of the algorithm in an unordered environment, such as urban streets where there are unpredictable pedestrians and vehicles, a city street scene with randomly crossing pedestrians, lane-changing vehicles, and intersections for cross-traffic is constructed in CARLA. Navigation performance tests are conducted for each algorithm, and the results are shown in Table 10.

**Table 10:** Comparison of navigation performance of various algorithms in chaotic urban street environments.

Algorithm type	NSR (%)	Average decision delay (s)	Path deviation rate (%)	Fault recovery time (s)
Improved REINFORCE	78.6	0.97	4.8	1.5
REINFORCE	70.2	1.12	5.9	2.8
DDPG	62.5	1.25	6.5	3.3
DQN	60.1	1.28	7.2	3.7
PPO	75.3	1.05	5.2	1.8
SAC	73.8	1.09	5.5	2.1

In Table 10, the improved REINFORCE algorithm still maintains the best navigation performance in the disordered urban street environment. Due to the random behaviors of dynamic targets and the cross-interference among multiple entities, the performance of each algorithm has decreased compared to the controlled environment. However, the NSR, decision-making speed, and fault recovery ability of this algorithm are still significantly superior to those of the other comparison algorithms.

## 5. Conclusion

Currently, VN technology still faces problems such as insufficient environmental perception and decision delay, which limit its application effectiveness in practical scenarios. This study proposed a REINFORCE algorithm based on policy gradient optimization, and combined ACA, ResBlock structure, and AM to construct an efficient VN model. It aimed to improve the navigation performance of ADR in complex environments. In the experiment, the improved algorithm achieved an NSR of 92.5% in the simulated environment, which was 15.8% higher than traditional methods. In real-world testing, the NDT of the robot was reduced by 20.3%, demonstrating good adaptability and efficiency. In addition, the algorithm also performed well in ACC and recall, reaching 95.7% and 91.2%, significantly better than other algorithms. The improved algorithm adopted by introducing ACA effectively enhanced the convergence speed and running efficiency. However, in real-world scenarios, although the NSR was high, the path deviation rate still needed to be further reduced, making it difficult to achieve more accurate path planning. In addition, the robustness of the algorithm when facing extremely complex environments still needs further verification. Future research will further optimize the algorithm structure, improve the adaptability and robustness of the model in complex environments, and explore more application scenarios to verify its wide applicability.

## Supporting information

S1 File Minimal Data Set. (DOC)

## References

[1] Pou B, Smith J, Quinones E, Martin M, Gratadour D. Integrating supervised and reinforcement learning for predictive control with an unmodulated pyramid wavefront sensor for adaptive optics. Opt Express. 2024;32(21):37011–35. doi: 10.1364/OE.530254 39573576

[2] Chen X, Yongchareon S, Knoche M. A review on computer vision and machine learning techniques for automated road surface defect and distress detection. J Smart Cities Soc. 2023;1(4):259–75. doi: 10.3233/scs-230001

[3] Bhattacharyya A, Nambiar SM, Ojha R, Gyaneshwar A, Chadha U, Srinivasan K. Machine Learning and Deep Learning powered satellite communications: Enabling technologies, applications, open challenges, and future research directions. Satell Commun Network. 2023;41(6):539–88. doi: 10.1002/sat.1482

[4] Kulsoom F, Narejo S, Mehmood Z, Chaudhry HN, Butt A, Bashir AK. A review of machine learning-based human activity recognition for diverse applications. Neural Comput Appl. 2022;34(21):18289–324. doi: 10.1007/s00521-022-07665-9

[5] Sajjan 5 M, Li J, Selvarajan R, Sureshbabu SH, Kale SS, Gupta R, et al. Quantum machine learning for chemistry and physics. Chem Soc Rev. 2022;51(15):6475–573. doi: 10.1039/D2CS00203E 35849066

[6] Song Y, Scaramuzza D. Policy Search for Model Predictive Control With Application to Agile Drone Flight. IEEE Trans Robot. 2022;38(4):2114–30. doi: 10.1109/tro.2022.3141602

[7] Jones M, Djahel S, Welsh K. Path-Planning for Unmanned Aerial Vehicles with Environment Complexity Considerations: A Survey. ACM Comput Surv. 2023;55(11):1–39. doi: 10.1145/3570723

[8] Li Z, Zhou A. RDDRL: A recurrent deduction deep reinforcement learning model for multimodal vision-robot navigation. Appl Intell. 2023;53(20):23244–70. doi: 10.1007/s10489-023-04754-7

[9] Yue P, Xin J, Zhang Y, Lu Y, Shan M. Semantic-Driven Autonomous Visual Navigation for Unmanned Aerial Vehicles. IEEE Trans Ind Electron. 2024;71(11):14853–63. doi: 10.1109/tie.2024.3363761

[10] Xie Z, Dames P. DRL-VO: Learning to Navigate Through Crowded Dynamic Scenes Using Velocity Obstacles. IEEE Trans Robot. 2023;39(4):2700–19. doi: 10.1109/tro.2023.3257549

[11] Yeom K. Deep Reinforcement Learning Based Autonomous Driving with Collision Free for Mobile Robots. Int J Mech Eng Robot Res. 2022;11(5):338–44. doi: 10.18178/ijmerr.11.5.338-344

[12] Assemlali H, Bouhsissin S, Sael N. Deep learning-driven CNN model for detection and classification of dynamic obstacles. Green Energy Intell Transp. 2025;100334. doi: 10.1016/j.geits.2025.100334

[13] Yahi N, Matute J, Karimoddini A. Receding horizon based collision avoidance for UAM aircraft at intersections. Green Energy Intell Transp. 2024;3(6):100205. doi: 10.1016/j.geits.2024.100205

[14] Wang H, Zhang G, Cao H, Hu K, Wang Q, Deng Y, et al. Geometry-aware 3D point cloud learning for precise cutting-point detection in unstructured field environments. J Field Robot. 2025;42(7):3063–76. doi: 10.1002/rob.22567

[15] Da C, Qian Y, Zeng J, Wei X, Zhang F. ST-PPO: a spatio-temporal attention enhanced proximal policy optimization algorithm for autonomous driving in complex traffic scenarios. Mach Learn. 2025;114(11):1–29. doi: 10.1007/s10994-025-06887-x

[16] Guo J, Zhou G, Huang H, Huang C. Advancements in UAV path planning: A deep reinforcement learning approach with soft actor-critic for enhanced navigation. Unmanned Syst. 2025;13(04):1065–84. doi: 10.1142/S2301385025500669

[17] Xie W, Jiang H, Zhu Y, Qian J, Xie J. Naviformer: A spatio-temporal context-aware transformer for object navigation. Proc AAAI Conf Artif Intell. 2025;39(14):14708–16. doi: 10.1609/aaai.v39i14.33612

[18] Botifoll M, Pinto-Huguet I, Arbiol J. Machine learning in electron microscopy for advanced nanocharacterization: current developments, available tools and future outlook. Nanoscale Horiz. 2022;7(12):1427–77. doi: 10.1039/D2NH00377E 36239693

[19] Baek JW, Chung K. Meta learning based object tracking technology: A survey. KSII Trans Internet Inf Syst. 2024;18(8):2067–81. doi: 10.3837/tiis.2024.08.001

[20] Zhao J, Hu H, Han Y, Cai Y. A review of unmanned vehicle distribution optimization models and algorithms. J Traffic Transp Eng (Engl Ed). 2023;10(4):548–59. doi: 10.1016/j.jtte.2023.07.002

[21] Xin J, Li Z, Zhang Y, Li N. Efficient real-time path planning with self-evolving particle swarm optimization in dynamic scenarios. Unmanned Syst. 2024;12(2):215–26. doi: 10.1142/S230138502441005X

[22] Pou B, Ferreira F, Quinones E, Gratadour D, Martin M. Adaptive optics control with multi-agent model-free reinforcement learning. Opt Express. 2022;30(2):2991–3015. doi: 10.1364/OE.444099 35209428

[23] Canese L, Cardarilli GC, Di Nunzio L, Fazzolari R, Giardino D, Re M, et al. Multi-agent reinforcement learning: A review of challenges and applications. Appl Sci. 2021;11(11):4948–73. doi: 10.3390/app11114948

[24] Wong A, Bäck T, Kononova AV, Plaat A. Deep multi-agent reinforcement learning: challenges and directions. Artif Intell Rev. 2023;56(6):5023–56. doi: 10.1007/s10462-022-10299-x

[25] Khochare A, Sorbelli FB, Simmhan Y, Das SK. Improved algorithms for co-scheduling of edge analytics and routes for UAV fleet missions. IEEE/ACM Trans Netw. 2024;32(1):17–33. doi: 10.1109/TNET.2023.3277810

[26] Saha SS, Sandha SS, Aggarwal M, Wang B, Han L, DE Gortari Briseno J, et al. TinyNS: Platform-Aware Neurosymbolic Auto Tiny Machine Learning. ACM Trans Embed Comput Syst. 2024;23(3):43. doi: 10.1145/3603171 38933471 PMC11200268

[27] Jeon H-M, Lim J-W, Ryoo C. Task Assignment for Multiple Multi-purpose Unmanned Aerial Vehicles Using Greedy Algorithm. Int J Aeronaut Space Sci. 2024;25(4):1380–94. doi: 10.1007/s42405-024-00726-4

[28] Avramov-Zamurovic S, Esposito JM, Nelson C. Classifying beams carrying orbital angular momentum with machine learning: tutorial. J Opt Soc Am A Opt Image Sci Vis. 2023;40(1):64–77. doi: 10.1364/JOSAA.474611 36607076

[29] Luo Q, Wu G, Trivedi A, Hong F, Wang L, Srinivasan D. Multi-Objective Optimization Algorithm With Adaptive Resource Allocation for Truck-Drone Collaborative Delivery and Pick-Up Services. IEEE Trans Intell Transport Syst. 2023;24(9):9642–57. doi: 10.1109/tits.2023.3267103

[30] Soares JL, Costa TB, Moura LS, Sousa WS, Mesquita AL, Braga DS. Fault diagnosis of belt conveyor idlers based on gradient boosting decision tree. Int J Adv Manuf Technol. 2024;132(7):3479–88.

[31] Zhou H, Jiang K, He S, Min G, Wu J. Distributed Deep Multi-Agent Reinforcement Learning for Cooperative Edge Caching in Internet-of-Vehicles. IEEE Trans Wireless Commun. 2023;22(12):9595–609. doi: 10.1109/twc.2023.3272348

[32] Zhang Y, Yang Q, An D, Li D, Wu Z. Multistep Multiagent Reinforcement Learning for Optimal Energy Schedule Strategy of Charging Stations in Smart Grid. IEEE Trans Cybern. 2023;53(7):4292–305. doi: 10.1109/TCYB.2022.3165074 35476564

[33] Suma M, Premananda R, Harakannanavar SS, Zhou W, Ji R, Lai J. Development of object recognition. Multimedia Tools Appl. 2023;82(28):43903–22. doi: 10.1007/s11042-023-14945-6

[34] Yun H, Kim E, Kim DM, Park HW, Jun MB-G. Machine Learning for Object Recognition in Manufacturing Applications. Int J Precis Eng Manuf. 2023;24(4):683–712. doi: 10.1007/s12541-022-00764-6

[35] Saha A, Rajak S, Saha J, Chowdhury C. A survey of machine learning and meta-heuristics approaches for sensor-based human activity recognition systems. J Ambient Intell Humaniz Comput. 2024;15(1):29–56. doi: 10.1007/s12652-022-03870-5

[36] Dornelas RS, Lima DA. Correlation Filters in Machine Learning Algorithms to Select Demographic and Individual Features for Autism Spectrum Disorder Diagnosis. J Data Sci Intell Syst. 2023;1(2):105–27. doi: 10.47852/bonviewjdsis32021027

