# Supplementary material for: Visual navigation technology for autonomous driving robots based on strategic gradient-REINFORCE algorithm
Source: PLoS One. 2026 May 11;21(5):e0347775. doi: 10.1371/journal.pone.0347775 (PMC13160307; doi:10.1371/journal.pone.0347775)
Supplement: S1 File — (DOC) [file pone.0347775.s001.doc]

**Figure 8. Comparison of ACC and recall rates of several algorithms**

(a) Comparison of accuracy rates

| Number of iterations | Improved REINFORCE | REINFORCE | DDPG | DQN |
| --- | --- | --- | --- | --- |
| 0 | 0 | 0 | 0 | 0 |
| 20 | 0.30 | 0.05 | 0.10 | 0.05 |
| 40 | 0.55 | 0.20 | 0.30 | 0.10 |
| 60 | 0.65 | 0.40 | 0.45 | 0.20 |
| 80 | 0.72 | 0.55 | 0.55 | 0.35 |
| 100 | 0.73 | 0.65 | 0.62 | 0.50 |
| 150 | 0.76 | 0.72 | 0.68 | 0.58 |
| 200 | 0.79 | 0.71 | 0.68 | 0.62 |
| 250 | 0.81 | 0.70 | 0.69 | 0.60 |
| 300 | 0.95 | 0.69 | 0.68 | 0.63 |
| 350 | 0.95 | 0.70 | 0.69 | 0.67 |
| 400 | 0.95 | 0.72 | 0.69 | 0.65 |
| 450 | 0.95 | 0.72 | 0.69 | 0.66 |
| 500 | 0.95 | 0.72 | 0.69 | 0.69 |

(b) Comparison of recall rates

| Number of iterations | Improved REINFORCE | REINFORCE | DDPG | DQN |
| --- | --- | --- | --- | --- |
| 0 | 0 | 0 | 0 | 0 |
| 20 | 0.30 | 0.10 | 0.08 | 0.05 |
| 40 | 0.55 | 0.25 | 0.20 | 0.15 |
| 60 | 0.70 | 0.40 | 0.35 | 0.30 |
| 80 | 0.85 | 0.55 | 0.50 | 0.45 |
| 100 | 0.84 | 0.70 | 0.62 | 0.55 |
| 150 | 0.85 | 0.73 | 0.70 | 0.62 |
| 200 | 0.85 | 0.76 | 0.73 | 0.64 |
| 250 | 0.85 | 0.75 | 0.74 | 0.63 |
| 300 | 0.86 | 0.74 | 0.75 | 0.66 |
| 350 | 0.87 | 0.74 | 0.76 | 0.67 |
| 400 | 0.91 | 0.75 | 0.77 | 0.68 |
| 450 | 0.91 | 0.75 | 0.77 | 0.66 |
| 500 | 0.91 | 0.75 | 0.77 | 0.71 |

**Figure 9. Comparison of convergence and runtime of several algorithms**

(a) Convergence case

| Number of iterations | Improved REINFORCE | REINFORCE | DDPG | DQN |
| --- | --- | --- | --- | --- |
| 0 | 0 | 0 | 0 | 0 |
| 20 | 0.15 | 0.08 | 0.06 | 0.04 |
| 40 | 0.60 | 0.30 | 0.35 | 0.32 |
| 60 | 0.85 | 0.45 | 0.50 | 0.40 |
| 80 | 0.90 | 0.58 | 0.55 | 0.45 |
| 100 | 0.95 | 0.60 | 0.60 | 0.50 |
| 150 | 0.90 | 0.62 | 0.62 | 0.55 |
| 200 | 0.96 | 0.65 | 0.66 | 0.56 |
| 250 | 0.94 | 0.64 | 0.63 | 0.55 |
| 300 | 0.95 | 0.67 | 0.68 | 0.57 |
| 350 | 0.96 | 0.72 | 0.70 | 0.56 |
| 400 | 0.97 | 0.75 | 0.69 | 0.53 |
| 450 | 0.96 | 0.77 | 0.68 | 0.51 |
| 500 | 0.93 | 0.76 | 0.66 | 0.45 |

(b) Run time (s)

| Number of iterations | Improved REINFORCE | REINFORCE | DDPG | DQN |
| --- | --- | --- | --- | --- |
| 0 | 3.20 | 6.40 | 8.10 | 20 |
| 20 | 5.30 | 9.20 | 14.30 | 27.80 |
| 40 | 6.50 | 11.40 | 16.70 | 32.60 |
| 60 | 7.60 | 12.10 | 17.50 | 31.20 |
| 80 | 8.20 | 12.80 | 18.90 | 29.40 |
| 100 | 8.70 | 13.60 | 20.20 | 30.10 |
| 150 | 9.10 | 14.30 | 21.40 | 32.80 |
| 200 | 10.20 | 15.20 | 22.30 | 37.50 |
| 250 | 10.60 | 16.10 | 23.50 | 34.20 |
| 300 | 11.30 | 17.80 | 25.10 | 33.40 |
| 350 | 12.20 | 16.30 | 27.90 | 35.70 |
| 400 | 13.60 | 15.70 | 30.20 | 41.80 |
| 450 | 14.10 | 18.90 | 32.40 | 43.20 |
| 500 | 15.70 | 19.40 | 34.80 | 44.90 |

**Figure 10. Comparison of RMSE and MAE of several algorithms**

(a) RMSE

| Number of iterations | Improved REINFORCE | REINFORCE | DDPG | DQN |
| --- | --- | --- | --- | --- |
| 0 | 0.110 | 0.150 | 0.140 | 0.130 |
| 20 | 0.100 | 0.110 | 0.120 | 0.120 |
| 40 | 0.097 | 0.110 | 0.110 | 0.110 |
| 60 | 0.098 | 0.109 | 0.105 | 0.110 |
| 80 | 0.097 | 0.105 | 0.103 | 0.107 |
| 100 | 0.096 | 0.103 | 0.102 | 0.106 |
| 120 | 0.098 | 0.102 | 0.102 | 0.105 |

(b) MAE

| Number of iterations | Improved REINFORCE | REINFORCE | DDPG | DQN |
| --- | --- | --- | --- | --- |
| 0 | 0.105 | 0.112 | 0.123 | 0.138 |
| 20 | 0.098 | 0.107 | 0.111 | 0.130 |
| 40 | 0.095 | 0.103 | 0.109 | 0.127 |
| 60 | 0.095 | 0.102 | 0.108 | 0.123 |
| 80 | 0.096 | 0.102 | 0.106 | 0.120 |
| 100 | 0.095 | 0.103 | 0.107 | 0.117 |
| 120 | 0.094 | 0.102 | 0.108 | 0.116 |

**Figure 11. Accuracy of the algorithm in the value function and the policy function**

(a) Value function

| Number of iterations | Improved REINFORCE | REINFORCE | DDPG | DQN |
| --- | --- | --- | --- | --- |
| 0 | 0.52 | 0.46 | 0.38 | 0.27 |
| 50 | 0.54 | 0.48 | 0.40 | 0.29 |
| 100 | 0.57 | 0.51 | 0.42 | 0.31 |
| 150 | 0.63 | 0.56 | 0.44 | 0.33 |
| 200 | 0.71 | 0.62 | 0.47 | 0.35 |
| 250 | 0.76 | 0.65 | 0.49 | 0.37 |
| 300 | 0.82 | 0.68 | 0.51 | 0.39 |
| 350 | 0.84 | 0.71 | 0.52 | 0.40 |
| 400 | 0.87 | 0.74 | 0.53 | 0.41 |
| 450 | 0.91 | 0.76 | 0.53 | 0.42 |
| 500 | 0.94 | 0.77 | 0.52 | 0.43 |

(b) Policy function

| Number of iterations | Improved REINFORCE | REINFORCE | DDPG | DQN |
| --- | --- | --- | --- | --- |
| 0 | 0.47 | 0.53 | 0.49 | 0.35 |
| 50 | 0.49 | 0.54 | 0.50 | 0.37 |
| 100 | 0.52 | 0.56 | 0.52 | 0.39 |
| 150 | 0.58 | 0.62 | 0.55 | 0.42 |
| 200 | 0.65 | 0.68 | 0.58 | 0.46 |
| 250 | 0.71 | 0.72 | 0.60 | 0.49 |
| 300 | 0.78 | 0.75 | 0.63 | 0.52 |
| 350 | 0.81 | 0.79 | 0.65 | 0.54 |
| 400 | 0.84 | 0.82 | 0.67 | 0.56 |
| 450 | 0.88 | 0.83 | 0.68 | 0.57 |
| 500 | 0.92 | 0.84 | 0.69 | 0.58 |

**Figure 12. Average reward value of VN of autonomous robot in simulated environment and real scene**

(a) The average reward value in simulated environment

| Data volume | Sliding average reward | Actual reward |
| --- | --- | --- |
| 0 | 200 | 390 |
| 200 | 220 | 320 |
| 400 | 280 | 380 |
| 600 | 450 | 460 |
| 800 | 550 | 620 |
| 1000 | 580 | 710 |
| 1200 | 590 | 650 |
| 1500 | 600 | 680 |
| 1800 | 605 | 720 |
| 2000 | 602 | 690 |
| 2500 | 598 | 670 |
| 3000 | 595 | 700 |

(b) The average reward value in real scenarios

| Data volume | Sliding average reward | Actual reward |
| --- | --- | --- |
| 0 | 200 | 280 |
| 200 | 230 | 310 |
| 400 | 320 | 390 |
| 600 | 520 | 580 |
| 800 | 610 | 650 |
| 1000 | 625 | 680 |
| 1200 | 630 | 660 |
| 1500 | 635 | 690 |
| 1800 | 632 | 670 |
| 2000 | 638 | 710 |
| 2500 | 636 | 680 |
| 3000 | 633 | 660 |
